# Modeling Solid State Stability for Speciation: A Ten-Year Long Study

**DOI:** 10.3390/molecules24163013

**Published:** 2019-08-20

**Authors:** Roberta Risoluti, Giuseppina Gullifa, Elena Carcassi, Francesca Buiarelli, Li W. Wo, Stefano Materazzi

**Affiliations:** 1 Department of Chemistry, “Sapienza” University of Rome, p.le A.Moro 5, 00185 Roma, Italy; 2 Department of Chemistry, Illinois State University, 100 N University St, Normal, IL 61761, USA

**Keywords:** speciation, biomimetic complexes, evolved gas analysis, TI-EGA-MS

## Abstract

Speciation studies are based on fundamental models that relate the properties of biomimetic coordination compounds to the stability of the complexes. In addition to the classic approach based on solution studies, solid state properties have been recently proposed as supporting tools to understand the bioavailability of the involved metal. A ten-year long systematic study of several different complexes of imidazole substituted ligands with transition metal ions led our group to the definition of a model based on experimental evidences. This model revealed to be a useful tool to predict the stability of such coordination complexes and is based on the induced behavior under thermal stress. Several different solid state complexes were characterized by Thermally Induced Evolved Gas Analysis by Mass Spectrometry (TI-EGA-MS). This hyphenated technique provides fundamental information to determine the solid state properties and to create a model that relates stability to coordination. In this research, the model resulting from our ten-year long systematic study of complexes of transition metal ions with imidazole substituted ligands is described. In view of a systematic addition of information, new complexes of Cu(II), Zn(II), or Cd(II) with 2-propyl-4,5-imidazoledicarboxylic acid were precipitated, characterized, and studied by means of Thermally Induced Evolved Gas Analysis performed by mass spectrometry (TI-EGA-MS). The hyphenated approach was applied to enrich the information related to thermally induced steps, to confirm the supposed decomposition mechanism, and to determine the thermal stability of the studied complexes. Results, again, allowed supporting the theory that only two main characteristic and common thermally induced decomposition behaviors join the imidazole substituted complexes studied by our group. These two behaviors could be considered as typical trends and the model allowed to predict coordination behavior and to provide speciation information.

## 1. Introduction

Speciation studies can be related by setting up fundamental models based on properties of biomimetic coordination compounds that provide the stability of the complexes in order to understand the bioavailability of the involved metal. 

Speciation models are mainly based on the classic approach by studies in different solution conditions. It is also well known that the thermal stability of a complex in the solid state is inversely proportional to the stability of the same complex in aqueous solution.

Metal complexes containing imidazoledicarboxylate ligands have been extensively studied because of their interesting properties. They are recognized to be realistic models as biomimetic simulators because of their characteristics, such as versatile structures useful for flexible tailoring. Additional interest has been demonstrated because of promising applications in gas storage, catalysis, optoelectronics, sensors, magnetism, luminescence, environment, and porous materials [1,2,3,4,5,6,7,8,9,10,11,12,13,14,15,16,17,18,19,20,21,22,23,24,25,26,27,28,29,30,31,32,33]. In consulting the literature, it is usual to find characterizations of new coordination compounds or complexes that report the synthesis, the elemental analysis, IR spectroscopic, and sometimes NMR or X-ray resulting information. More frequently, additional information is obtained from the solid state precipitates by means of thermal behavior. It is now globally recognized that the thermal stress induced on the complexes is able to provide kinetic and chemical decomposition information of the examined samples. This approach in itself is not sufficient to explain complex releasing steps. The most recent approach on-line couples FTIR or MS spectroscopies to increase the information and correctly characterizes the releasing (or decomposition) steps. The obtained data from the Thermally Induced Evolved Gas Analysis (TI-EGA) are becoming valuable supporting information that proposes a more complete characterization of the study of thermally induced decomposition mechanisms [34,35,36,37,38,39,40,41]. Our group reported these advantages in several reviews [42,43], enhancing the very different fields of application. This hyphenated approach is recognized as a very useful tool to propose decomposition mechanisms for precipitated complexes [44,45,46,47,48]. To step ahead, our group recently suggested new trends in thermal analysis [49,50,51,52,53,54,55] also comparing a new approach by portable microNIR, both oriented on the application of chemometrics [56,57].

A ten-year long systematic study of several different complexes of imidazole substituted ligands with transition metal ions led our group to the definition of a model based on experimental evidences. This model is revealed to be a useful tool to predict the stability of such coordination complexes and is based on the induced behavior under thermal stress. Several different solid state complexes were characterized by Thermally Induced Evolved Gas Analysis by Mass Spectrometry (TI-EGA-MS). This hyphenated technique provides fundamental information to determine the solid state properties and to create a model that relates stability to coordination.

The results of our ten-year long systematic study on several different complexes of substituted imidazole ligands with transition metal ions gave us the experimental evidence of two characteristic reproducible decomposition pathways. A predictive model was consequently proposed by our group. The TI-EGA-MS results allowed us to propose, for all these complexes, preliminary low-temperature thermally induced steps related to the loss of water molecules and counter ions, already present, followed by two different reproducible discriminating trends:

- The rupture of side chains, to give a five- or six-member ring as intermediate, compatibly with the percent weight loss and the TI-EGA-MS information. This behavior was recorded with ligands, such as *N*,*N*′-bis-(2-hydroxybenzylidene)-1,1-diaminobutane, 2-aminomethyl-benzimidazole, imidazole-4,5-dicarboxylic acid, and similar structures;
- The total loss of substitutions, with an imidazole 1:2 or 1:4 complex remaining as intermediate, before the last decomposition step involving the metal oxide. This behavior was recorded with ligands, such as (1-methylimidazol-2-yl)ketone, dopamine, and derived structures. All these studies are described in the references [57,58,59,60,61,62,63,64,65,66,67,68] and are the experimental evidences on which the proposed model is based. This thermally induced behavior, and the consequently derived model, is proposed as a tool to provide stability information on the complexes to be related to speciation studies.

The robustness of this predictive model needs additional examples to be continuously inserted. This study of new solid state complexes of Cu(II), Zn(II), and Cd(II) with 2-propyl-4,5-imidazoledicarboxylic acid was carried out with two main goals: i) To predict the stability from the solid state characteristics and ii) to add experimental evidences to the model.

Complexes were precipitated and characterized following previously reported procedure to be correctly compared. On the basis of the resulting characteristics, a predicted behavior and consequent stability was predicted by the model. The model prediction was successfully confirmed by the results of the Thermally Induced Mass Spectrometry Evolved Gas Analysis (TI-EGA-MS). Results, again, showed that between the two main common thermally induced decomposition behaviors, the one predicted by the model joined the substituted imidazole complexes studied by our group and could be considered as typical trends for these structures.

## 2. Results and Discussion

The results from the elemental analysis of the precipitated complexes are reported in Table 1. Calculated and experimentally measured element percents are in good agreement.

As for similar complexes, reported by Yang and coworkers [69], FTIR spectra confirmed the main common absorption band (KBr, cm^−1^): 2975 (m), 1720 (s), 1540 (s), 1390 (s), 1280 (s), 1100 (m), 860 (m), 775 (m), 695 (w), 660 (m), 510 (m).

The Solid State Model, on the basis of these characteristics, predicted these complexes belonging to the first group described in the introduction.

**Figure 1:**
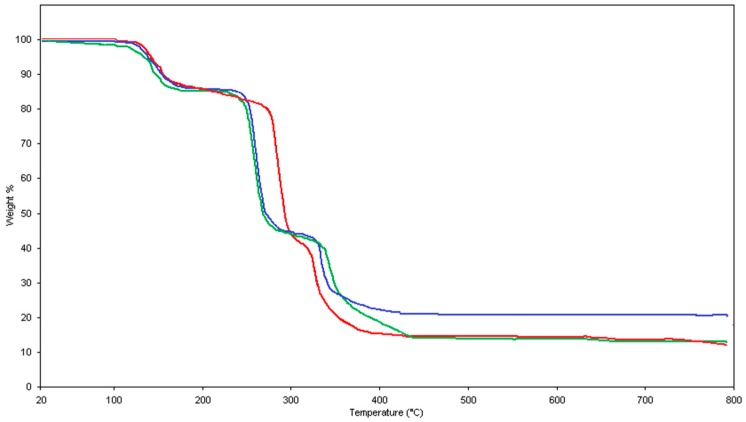
Thermally induced releasing profiles of the Cu(H_2_PIDC)_2_(H_2_O)_2_ (blue curve), Cd(H_2_PIDC)_2_(H_2_O)_2_ (red curve), and Zn(H_2_PIDC)_2_(H_2_O)_2_ (green curve): Air flow at 100 mL min^−1^; heating rate 5 °C min^−1^.

Thermally induced releasing steps of the precipitated Cu(H_2_PIDC)_2_(H_2_O)_2_, Zn(H_2_PIDC)_2_(H_2_O)_2_, and Cd(H_2_PIDC)_2_(H_2_O)_2_ were comparatively studied by thermally induced evolved gas analysis by mass spectrometry (TI-EGA-MS) to confirm the decomposition mechanism proposed by the model. In Figure 1, the thermoanalytical profiles of the three complexes, registered while heating the precipitates, are overlapped to compare the releasing steps under the oxidant (air) purging flow.

As previously reported for similar complexes, the thermally induced behavior was confirmed to be based on three main steps (see Table 1) with a first release of the water molecules and of only one side chain of the ligand. This hypothesis can be based on the molecular structure of this complex that shows one side chain in the external position, consequently easier to be removed.

**Figure 2:**
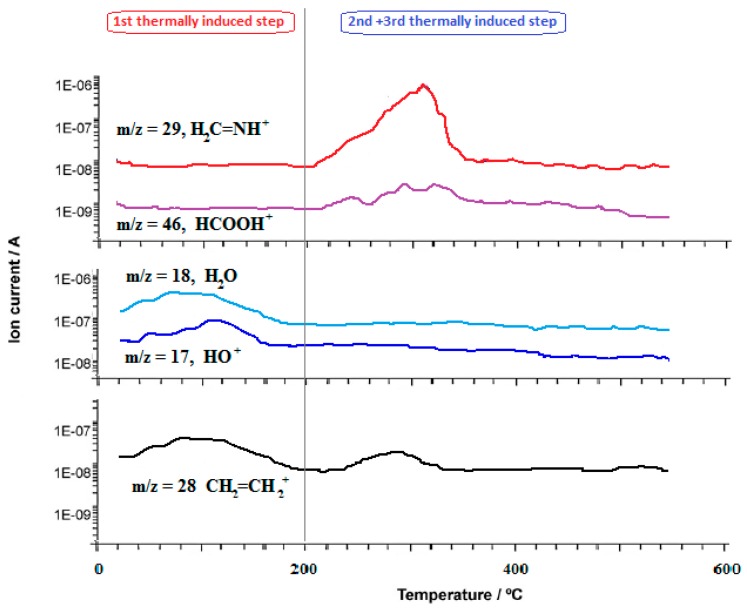
Representative curves of Thermally Induced Evolved Gas Analysis by Mass Spectrometry: *m*/*z* traces commonly recorded as a function of the temperature for all the analyzed complexes.

The consequent Evolved Gas Analysis by Mass Spectrometry confirmed the release of the two water molecules by detecting fragments at *m*/*z* = 17 and 18, and of the side chain by *m*/*z* = 28, in the temperature range of 100–200 °C, as shown in Figure 2. The behavior was not influenced when the oxidant flow (air) was changed to inert flow (N_2_).

**Figure 3:**
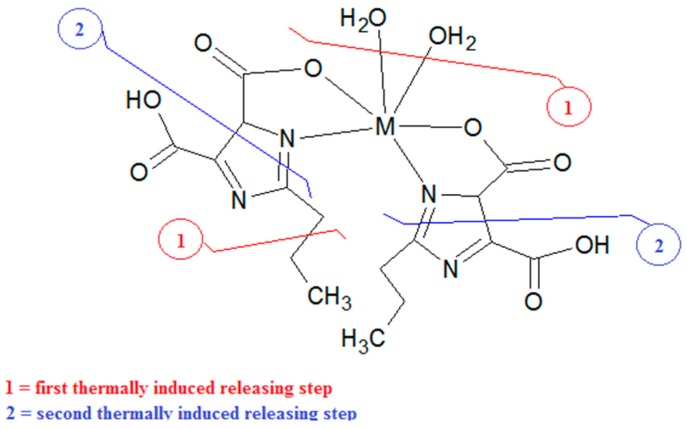
Scheme of the general decomposition mechanism.

In the second releasing process (200–300 °C), the presence of fragments at *m*/*z* = 28, 29, and 46 when nitrogen is the reacting flow (Figure 2) and the calculated percent weight loss, proved the rupture of the ligand ring, as depicted in Figure 3, and the temporary consequent rearrangement. The final third thermally induced step (300–500 °C) led to the complete decomposition of the residual compound to obtain the metal oxide.

By matching the MS fragmentation and the correspondence between percent weight loss calculated and percent weight loss experimentally recorded (Table 2), the proposed decomposition mechanism is clearly supported.

The thermal behavior of the complexes was also verified by an inert purging flow (nitrogen) to check the differences when the pyrolysis took place instead of oxidation. Only the final step showed a different shape due to the uncompleted reaction to give the metal oxides.

No effects due to the inert atmosphere were detected up to 300 °C. 

Consequently, the results clearly showed that the studied complexes of transition metal ions with 2-propyl-4,5-imidazoledicarboxylic acid belong to the first group described in the introduction.

The model correctly predicted the corresponding group.

## 3. Experimental and Methods

### 3.1. Materials

The ligand 2-propyl-4,5-imidazoledicarboxylic acid (H_3_PIDC) and the copper, zinc, and cadmium salts were purchased from Sigma-Aldrich-Merck Co. (St. Louis, MO, USA). All the reagents were of A.R. grade and used without further purification. The conditions already reported for the previously published similar complexes were strictly followed.

### 3.2. Instrumental

Elemental and spectroscopic analyses, thermoanalytical characterization, and consequent Thermally Induced Evolved Gas Analysis by Mass Spectrometry (TI-EGA-MS) were performed as previously reported [68,70,71].

## 4. Conclusions

This study of newly synthesized transition metal complexes is aimed to contribute to a larger systematic investigation to support the two-way characteristic decomposition path that is strictly related to the structural stability of the precipitated complexes.

The ten-year long based model correctly predicted the characteristics of the precipitated complexes, anticipating what was experimentally confirmed.

## Figures and Tables

**Table 1:** Elemental analysis results for the precipitated complexes. Cu, Zn, or Cd (Metal%) were determined by ICP-OES.

Complex	C/%			H/%			N/%			Metal/%	
	Found	Calculated		Found	Calculated		Found	Calculated		Found	Calculated
Cu(H_2_PIDC)_2_(H_2_O)_2_	39.1	39.3		4.7	4.5		11.8	11.4		12.2	12.1
Zn(H_2_PIDC)_2_(H_2_O)_2_	39.3	39.3		4.7	4.5		11.5	11.4		11.9	12.1
Cd(H_2_PIDC)_2_(H_2_O)_2_	27.2	27.4		4.5	4.0		7.9	8.0		21.1	21.3

**Table 2:** Temperature range of the main thermal steps and the corresponding percent weight loss.

Complex	First TG Step 100–190 °C Weight Loss %			Second TG Step 230–300 °C Weight Loss %			Third TG Step 300–450 °C Weight Loss %	
	Found	Calculated		Found	Calculated		Found	Calculated
Cu(H_2_PIDC)_2_(H_2_O)_2_	13.3	13.1		45.0	45.8		25.7	26.0
Zn(H_2_PIDC)_2_(H_2_O)_2_	13.0	13.1		46.9	45.8		24.0	26.0
Cd(H_2_PIDC)_2_(H_2_O)_2_	11.6	11.2		43.0	43.3		24.3	24.2
